# Perspectives of parents receiving normal results from genomic newborn screening: a mixed-methods evaluation from the early check program

**DOI:** 10.3389/fgene.2025.1704364

**Published:** 2025-12-03

**Authors:** Angela Y. Gwaltney, Sean N. Halpin, Samantha Scott, Sara M. Andrews, Katherine C. Okoniewski, Heidi L. Cope, Melissa Raspa, Curt Scharfe, Holly Peay

**Affiliations:** 1 Center for Informatics, RTI International, Research Triangle Park, Durham, NC, United States; 2 GenOmics and Translational Research Center, RTI International, Research Triangle Park, Durham, NC, United States

**Keywords:** informed consent, electronic consent, genomic newborn screening, whole genome sequencing, participant attitude, mixed-methods evaluation, public health genomics, health literacy

## Abstract

As genomic technologies become increasingly practicable for public health application, research programs are exploring population-scale genomic newborn screening (gNBS). *Early Check* is a statewide newborn screening research program in North Carolina that offers optional whole genome sequencing-based screening to parents of newborns through an electronic education, consent, and return of results platform. Parents can elect to receive screening for over 200 monogenic conditions and risk for type 1 diabetes (T1D) using a genetic risk score (GRS). To address knowledge gaps in gNBS implementation, we conducted a concurrent mixed-methods study evaluating the feasibility, acceptability, and effectiveness of the program’s electronic education, consent, and return of results processes among participants who received normal screening results. We emailed the evaluation survey link to consenting parents of all participating newborns with screening results showing no increased risk identified (n = 3,496). Survey respondents could indicate their willingness to participate in an interview. A total of 279 surveys and 14 interviews were included for quantitative and qualitative analysis. Findings revealed high levels of satisfaction (94.8% positive attitude). Overall, levels of regret were low, with two-thirds of respondents reporting no regret and an additional quarter reporting mild regret. Nonetheless, the proportion reporting mild regret indicates that a meaningful subset experienced some degree of uncertainty or second thoughts. Additionally, there was generally moderate comprehension overall, with 60.9% of parents demonstrating adequate knowledge when assessed across key items. Parents’ frequently-endorsed motivators included learning about their baby’s future health, the study being free, and the study not requiring extra blood samples. Although most appreciated the convenience and clarity of the process, some requested more information on topics including genetic testing and T1D GRS. Parents reported limited use of the educational videos and laboratory-generated screening reports, but most viewed the lay summary of normal results. Despite this selective engagement, they described the overall experience as accessible and well-designed. Parents’ decisions to share results were primarily confined to family members, with fewer disclosures to healthcare providers. This study supports the feasibility and acceptability of large-scale electronic consent and return of results processes but highlights some challenges in comprehension and equity across diverse populations.

## 1 Introduction

The aim of newborn screening (NBS) is to identify serious but treatable conditions soon after birth (CDC, 2023). Apart from point-of-care screening, the standard protocol involves analyzing a small blood sample to detect conditions which, if left untreated, may result in severe morbidity or mortality. In the United States, each state determines its own screening panel, though most align with the federally recommended 38 conditions on the Recommended Uniform Screening Panel (CDC, 2023). NBS programs primarily use biochemical assays to detect a defined set of metabolic and genetic conditions, though many also incorporate molecular methods for confirmation or for screening certain disorders. However, the current scope of NBS is limited by multiple factors, including available treatments, test characteristics, and public health implementation logistics. As a result, many treatable conditions remain undetected through traditional NBS.

In contrast, next-generation sequencing (NGS) modalities, which analyze an individual’s genetic code, including whole exome sequencing and whole genome sequencing, analyze part or all of an individual’s genetic code and enables identification of a much broader range of conditions than traditional NBS methods (Schobers et al., 2024). NGS facilitates earlier opportunities for care for additional conditions by identifying genetic variants linked to future health risks, supporting proactive clinical planning (Jeanne and Chung, 2025; Kansal, 2025). By expanding the range of detectable conditions, NGS has the potential to revolutionize NBS and improve health outcomes (Rehm, 2025). Historically, however, widespread implementation of NGS has been limited by high costs and restricted access to sequencing technologies (Messner et al., 2017). As sequencing technologies become more affordable and accessible, conversations have shifted from feasibility to practical and ethical considerations for implementation in public health programs, particularly NBS (Satam et al., 2023; Milko and Khoury, 2022). Declining costs now make population-scale genomic newborn screening (gNBS) a realistic possibility, prompting renewed attention to equity, trust, and the accessibility of education and consent processes (Milko and Khoury, 2022). Alongside technological advances, improved bioinformatics and curated population databases (e.g., gnomAD) have enhanced variant interpretation (Shah et al., 2024). However, the prevalence of variants of uncertain significance (VUS) creates persistent challenges; expert curation and functional follow-up remain essential before responsibly deploying genomic screening at population scale.

Despite advances in technology and cost reduction, implementing gNBS raises ongoing challenges related to feasibility and public acceptance. Integrating sequencing into public health programs not only requires logistical planning but also attention to public perceptions, trust, and values (Milko and Khoury, 2022). To ensure broad benefit, education and consent materials must be designed to be accessible, inclusive, and culturally responsive (Milko and Khoury, 2022).

As shown in Armstrong et al. (2022), parental attitudes towards gNBS are generally positive but nuanced. While parents are supportive of learning actionable health information through sequencing, they also express a clear desire for thorough education, informed consent, and transparency about how genomic data will be used. These findings reinforce the importance of designing gNBS programs that are ethically sound, respect parental autonomy, and are responsive to the informational needs and preferences of families.

*Early Check* is a pioneering newborn screening research program in North Carolina, established in 2018 to collect evidence to inform NBS policy. The program obtains electronic consent from parents or legal guardians (typically the biological mother) for the newborn’s participation in research. *Early Check* initially offered supplemental screening for fragile X syndrome, spinal muscular atrophy, and Duchenne muscular dystrophy using more traditional testing modalities (Bailey et al., 2019; Okoniewski et al., 2019; Kucera et al., 2021; Kucera et al., 2024). In this initial phase of *Early Check*, parents could provide consent any time after the first trimester through 31 days after birth. An evaluation of this targeted screening found high acceptability, trust, and knowledge recall among surveyed and interviewed parents, with consistently positive attitudes toward screening (Peay et al., 2022).

In 2023, *Early Check* expanded its scope to incorporate whole genome sequencing (WGS) to screen for over 200 monogenic conditions, as well as risk for type 1 diabetes (T1D), calculated with a genetic risk score (GRS) (Cope et al., 2024). When the switch to WGS occurred, only postnatal consents–within 31 days after birth–were collected, due to a quality control requirement to confirm that the baby’s reported sex matched the genetic sex identified through sequencing. The aim of this study was to evaluate how parents of participants who received normal screening results perceived the educational content, consent process, and return of results experience, with particular attention to informed choice, trust, and understanding. This analysis focuses exclusively on parents of screen-negative participants, as the experiences of parents of screen-positive children differ substantially and will be presented in a separate publication.

## 2 Materials and methods

We applied a mixed-methods approach with iterative quantitative and qualitative analysis (Creswell and Creswell, 2017). Our goal in using mixed methods was to gain a fuller understanding of the experience of parents of *Early Check* participants who received normal screening results. The study was approved by the University of North Carolina Institutional Review board (IRB; #18-0009). Parents of enrolled newborns, generally the mother, were asked to participate in this evaluation study after the return of normal screening results for the newborn.

### 2.1 *Early Check* screening groups

Parents could consent to screening their newborn for three groups of conditions. Additional detail is available (Cope et al., 2024).

#### 2.1.1 Group 1 – Monogenic Screening for Treatable Conditions

All enrolled newborns were screened for ∼200 monogenic conditions for which early diagnosis and intervention can significantly improve outcomes or prevent serious symptoms. Conditions in this group are often severe or life-threatening without timely treatment. Examples include retinoblastoma, Jervell and Lange-Nielson syndrome, and hemophilia.

#### 2.1.2 Group 2 – Monogenic Screening for Conditions with potential treatment (optional screening)

Newborns could be screened for a smaller set of monogenic conditions (∼40) that currently have emerging treatments but may benefit from early identification. These conditions typically result in serious developmental or physical impairments. Examples include Rett syndrome and Duchenne muscular dystrophy. Early diagnosis may enable access to specialized care and opportunities to participate in research aimed at developing better treatments.

#### 2.1.3 Group 3 – Risk for type 1 diabetes (optional screening)

Parents were offered the option to learn about their child’s risk for developing T1D. Using a GRS, newborns are placed into one of four lifetime risk categories: Low concern (<2% risk), Moderate concern (≥2 to <5% risk), Higher concern 1 (≥5 to <10% risk), and Higher concern 2 (≥10% risk). This screening does not provide a definitive diagnosis but identifies level of risk, allowing families and clinicians to monitor for early signs of the condition, enabling earlier diagnosis and intervention.

### 2.2 *Early Check* participants

*Early Check* participants were newborns born in North Carolina, enrolled within 31 days of birth by their birthing parent or legal guardian. Families must reside in North Carolina or South Carolina, and the baby must have received standard North Carolina NBS. The primary method of recruitment was a letter addressed from the North Carolina State Laboratory of Public Health mailed to mothers of newborns who underwent NBS in the state (Paquin et al., 2021). At the time of consent, parents were asked to report the date of birth, sex, and race/ethnicity of the newborn as well as the date of birth, address, education, and preferred language of the consenting parent. Because education was not a required field on the consent form, this variable had high levels of missingness. Participant data collected through the consent portal were used to assess whether the survey sample was representative of the broader study population.

### 2.3 Return of results processes

When results were available in the *Early Check* portal, the consenting parent of participating newborns with normal screening results was notified via email (Cope et al., 2023). As described in the consent materials, if results were normal parents would receive an email notifying them that results could now be viewed in the portal. Parents who accessed the portal could view a high-level summary written in plain language that explained their newborn’s results were normal. We defined “normal results” as no reportable monogenic variants and low lifetime risk (<2% risk) for T1D. There were separate notification emails and results pages for monogenic and T1D screening results. On the T1D results page parents could watch educational videos explaining the screening process and what the low concern result meant. Parents could also schedule an educational session with a genetic counselor or contact the *Early Check* team with questions via phone or email. Parents then had the option to review or download the full laboratory-generated results report. We intentionally designed the return of results process so that parents did not need to engage with complex lab reports to understand their child’s screening outcome. The goal was to make the result clear and accessible through a user-friendly interface.

### 2.4 Survey

We asked parents of all *Early Check* participants who received normal screening results between 28 May 2024 and 6 May 2025, to complete an online questionnaire (Supplementary Material A). Recruitment emails were sent to the individual who consented their newborn into the program, typically the mother. The 43-item survey (Supplementary Material) was administered through REDCap (Research Electronic Data Capture; Harris, et al., 2009) and included both closed- and open-ended questions across multiple domains relevant to their experience with *Early Check*. The first batch of survey invites were sent on 28 August 2024, to parents who had received normal (negative) screening results between 28 May 2024 and that date. Thereafter, invitations were distributed weekly to parents whose infants received normal results during the preceding week. We drew on established frameworks to conceptualize informed choice as a combination of adequate knowledge, value-consistent attitudes, and corresponding behavior (Marteau et al., 2001; Michie et al., 2002). The questionnaire was designed to assess informed decision-making, understanding of the screening process, emotional responses to participation and willingness to engage in future research. Several survey items were adapted from existing validated measures, including harmonized measures aggregated by the Clinical Sequencing Evidence-Generating Research (CSER) consortium (Amendola et al., 2018). Demographic or background items asked included their education level, health literacy (via a validated single-item measure; Haun et al., 2009), and type of device used to review *Early Check* information. At the end of the survey, respondents were asked about their interest in participating in a follow-up qualitative interview.

#### 2.4.1 Reasons for participating in *Early Check*

Participants were asked to rank their top three reasons for enrolling their baby in *Early Check*, selecting from a list of practical (e.g., “It was free,” “It was easy to sign up”), personal (“e.g., “Peace of mind,” “Learning about my baby’s health”), and altruistic motivations (e.g., “Helping future babies,” “Supporting research”). These items were generated based on our evaluation of prior *Early Check* pilot studies (Peay et al., 2022). Those who chose to include T1D risk screening were presented with an additional list of motivations specific to that decision, such as a family history of diabetes, access to free follow-up testing, and knowing someone with T1D. A question for participants who declined T1D screening was added toward the end of data collection, asking them to select up to three reasons for opting out, including low concern about their child’s risk, concerns about test accuracy or follow-up care, and anxiety about receiving results.

#### 2.4.2 Perceptions and adequacy of information

Participants rated the clarity, trustworthiness, and usefulness of *Early Check* information for both general content and specific screening categories: Monogenic Screening for Treatable Conditions (Group 1) and Monogenic Screening for Conditions with Potential Treatments (Group 2). If the participant had selected T1D risk screening, they responded to these same items for T1D materials. A five-point Likert scale (Strongly disagree to Strongly agree) was used to rate statements such as “The information was easy to understand,” “The information was trustworthy,” and “The information helped me decide whether to enroll.”

To assess the adequacy of the information provided, respondents who had reviewed the relevant materials were asked whether more information would have been helpful across 13 topic areas, including the screening and enrollment process, genetic testing, risk estimation, T1D screening and genetic risk score (GRS) calculation, benefits and risks of participation, potential treatments, privacy and security, and the standard newborn screening. Response options include “No, no additional information was needed,” “Yes, some more information would have been helpful,” “Yes, a lot more information would have been helpful,” and “Not applicable to what I signed up for.”

#### 2.4.3 Knowledge of *Early Check*

Understanding of *Early Check* screening was assessed using a seven-item multiple-choice knowledge scale adapted from our prior *Early Check* evaluation (Peay et al., 2022). Each item offered “True,” “False,” and “Unsure” response options. Correct responses were scored as one point; incorrect and unsure responses were scored as zero. Total scores ranged from 0 to 7, with a score of five or higher considered to indicate good knowledge, consistent with previous studies (Michie et al., 2002; van den Berg et al., 2006).

During the final 3 months of survey collection, we added an exploratory item “Some babies with a ‘low concern’ result will still develop T1D during their lifetime” to assess understanding of residual risk. Due to limited responses, this item was not included in the total knowledge score.

#### 2.4.4 Attitudes toward participation

Attitudes toward *Early Check* participation were assessed using the five-item attitude scale adapted from the validated attitude component of the Multidimensional Measure of Informed Choice (MMIC) introduced by Michie et al. (2002), refined for prenatal screening by Lewis et al. (2016), and also used in *Early Check*’s prior evaluation (Peay et al., 2022). Respondents rated their agreement with five bipolar adjective pairs (e.g., beneficial-harmful, important-unimportant, a good thing = a bad thing, reassuring-not reassuring, and desirable-undesirable) on a five-point Likert scale. Scores ranged from 0 to 20 and were categorized as positive (0-6), neutral (7-13), or negative (14-20), following standard MMIC scoring procedures.

#### 2.4.5 Experience receiving results

Participants who accessed their baby’s screening results were asked about their experience reviewing, interpreting, and sharing the information. This included whether they viewed their baby’s results, whether they read accompanying explanatory materials, and whether they had or planned to discuss the results with others (e.g., healthcare providers, family). Respondents who opted into T1D screening received similar questions specific to T1D results, including use of educational videos and perceived understanding of the information. All participants were asked to rate the usability of the *Early Check* online portal.

#### 2.4.6 Decision regret

Decision regret was measured using the five-item Decision Regret Scale (DRS; Brehaut et al., 2003), which was also used in an earlier evaluation of the *Early Check* pilot study (Peay et al., 2022). Participants rated each item on a five-point Likert scale, with scores converted to a 0–100 scale, where 0 indicates no regret and 100 indicates maximum regret. For analysis, DRS scores were categorized into no regret (score of 0), mild regret (scores from 1 to 25), and high regret (scores greater than 25). These thresholds have been used in previous studies and are summarized in a systematic review of DRS applications (Becerra Pérez et al., 2016).

### 2.5 Interviews

All one-on-one interviews were conducted by researchers with qualitative methods expertise (SNH, SS) using an online video-conferencing platform, in alignment with best practices for designing and conducting qualitative interview studies (Roulston and Halpin, 2022). We used purposive sampling to select participants from among those who completed the survey and indicated willingness to participate in an interview. Our sampling strategy aimed to capture a range of participant perspectives, with intentional inclusion of racial and ethnic minority participants to better reflect the diversity of the broader *Early Check* population.

The semi-structured 26-item interview guide (Supplementary Material B included eight sections: (1) study overview and recruitment experiences, (2) consent portal review, (3) monogenic results experience, (4) monogenic results review, (5) T1D results experience, (6) T1D results review, (7) decision satisfaction, and (8) final thoughts. Throughout the interview, participants were prompted to recall their experiences consenting for *Early Check* and/or reviewing their screening results. Visual stimuli in the form of screenshots from the consent portal and results portal were used to support recall and elicit detailed responses.

Survey responses were used as contextual anchors for interview questions to deepen exploration of participant understanding. For example, “On the survey you answered the question ‘how well do you understand your baby’s test results?’ with [insert answer to question]. Looking at the sample results report now, can you tell me how easy or difficult it was to understand your baby’s normal results?”

Immediately after each interview, the interviewer wrote an analytic memo documenting key concepts, unexpected responses, and reflections on participant meaning-making (Emerson et al., 2011). These memos were shared between the qualitative researchers and served as an initial basis for developing early inductive codes and identifying potential themes.

### 2.6 Data analysis

#### 2.6.1 Quantitative analyses

Most quantitative analyses were conducted using SAS 8.3 (SAS Institute, Inc., Cary, NC). Descriptive statistics were calculated for all variables. Continuous variables (e.g., age) were summarized using means, standard deviations, and ranges whereas categorical variables were summarized with frequences and percentages. To compare the survey sample with the larger consented study population, independent samples *t*-tests were conducted for continuous variables and chi-square tests were applied for categorical variables. To address estimate stability, maternal education was collapsed into three levels: less than a bachelor’s degree, a bachelor’s degree, and more than a bachelor’s degree. Fisher’s exact tests were used when expected cell counts were less than 10. In cases of large sparsity (e.g., Race and Maternal Education variables), we used a Monte Carlo simulation approach to estimate the Fisher’s exact test p-value. The simulation was implemented in R (version 2024.12.1) using the “fisher.test ()” function with “simulate.p.value = TRUE” and 10,000 replicates to ensure adequate precision.

Differences in ordinal measures of clarity, trustworthiness, or helpfulness in decision making were examined by education level, health literacy, and preferred language using chi-square tests, with Fisher’s Exact test applied when cell sizes were small. A binomial regression was conducted to assess whether education level was associated with achieving adequate knowledge of the program, as measured by the knowledge scale.

Comprehension scores, rated on a five-point Likert scale (0 = *Not at all* to 4 = *Extremely well*) were compared across subgroups using ANOVA or the Kruskal–Wallis test, depending on sample size and data distribution. ANOVA results for education level comparisons were reported as means with standard deviations, whereas the Kruskal–Wallis test was used for comparisons by health literacy and preferred language with results presented as medians and interquartile ranges.

Differences in decision regret, categorized into three levels, were examined education level, race, and T1D risk screening status using Fisher’s exact test to account for small subgroup sizes. Attitudes toward the programs (positive, neutral, or negative) were compared between participants who opted for T1D risk screening and those who did not using chi-square tests.

Metadata on the language of the consent portal (English or Spanish) was captured automatically at the time of enrolment. Self-reported preferred language spoken at home was also collected through the *Early Check* portal, with response options including English, Spanish, or Other. As this was an optional question, for participants who did not provide a response, the language used during the electronic consent process (English or Spanish) was used as a proxy. Zip code was linked to the U.S. Department of Agriculture’s Rural-Urban Commuting Area (RUCA) codes, which were collapsed into three categories to represent urbanicity: urban areas with populations of 50,000 or more people, large rural cities or towns with populations between 10,000 and 49,999, and small and isolated towns with populations under 10,000 residents (U.S. Department of Agriculture Economic Research Service, 2020).

#### 2.6.2 Qualitative analyses

All qualitative data were managed in NVivo version 14.0 (Lumivero, United States) and analyzed using a combination of deductive and inductive thematic analysis (Nowell et al., 2017). We developed an initial codebook with deductive codes corresponding to the interview guide sections and key concepts of interest (e.g., consent experience, understanding of results, trust, motivations for participation, perceived clarity of materials). These included topics related to consent, understanding of results, trust, motivations for participation, and perceived clarity of educational content.

Subsequently, an inductive analytic phase was conducted to identify emergent themes from the interview data and analytic memos. Codes were refined and expanded collaboratively during team discussions. Rather than calculating inter-coder agreement, we employed a team-based reflexive approach to consensus coding, consistent with guidance that discourages use of inter-coder reliability metrics in interpretive qualitative research where coding reflects conceptual meaning rather than surface-level agreement (Halpin, 2024; Halpin, 2025). We enhanced the trustworthiness of the qualitative analysis by using multiple coders, maintaining an audit trail of memos and code revisions, and resolving differences through discussion (Nowell et al., 2017).

## 3 Results

### 3.1 Participant demographics and background characteristics

A total of 3,496 newborns received normal screening results during this time (Table 1). Of these, 279 mothers completed the survey (8% response rate) and 14 participated in qualitative interviews. Survey respondents were, on average, slightly older than mothers in the overall parent cohort (mean of about 34 vs. 32.5 years of age), a difference that was statistically significant (*p* < 0.0001). Interview participants were generally older than both the overall study population and the subset of participants who completed the survey.

**TABLE 1:** Participant demographics and background characteristics.

Characteristic	Newborns with normal screening results (n = 3217)	Survey respondents (n = 279)	p-value	Interviewees (n = 14)
Maternal age, years				
Mean (STD)	32.5 (5.1)	33.9 (4.5)	<0.0001	35.0 (4.7)
Min - Max	18–50	19–50		28–42
Baby’s sex			0.27	
Female	1618 (50.3%)	150 (53.8%)		10 (71.4%)
Male	1599 (49.7%)	129 (46.2%)		4 (28.6%)
Maternal Race/Ethnicity			0.0002	
African American/Black	346 (10.8%)	16 (5.7%)		2 (14.3%)
American Indian/Alaskan Native	24 (0.8%)	0 (0%)		0 (0%)
Asian	244 (7.6%)	10 (3.6%)		2 (14.3%)
Hispanic/Latino or Spanish	299 (9.3%)	15 (5.4%)		0 (0%)
Middle Easter/North African	20 (0.6%)	0 (0%)		0 (0%)
Native Hawaiian/Pacific islander	2 (0.1%)	0 (0%)		0 (0%)
White	1970 (61.2%)	210 (75.3%)		9 (64.3%)
Two or more races	272 (8.5%)	24 (8.6%)		1 (7.1%)
Unknown/Prefer not to respond	40 (1.2%)	4 (1.4%)		0 (0%)
Maternal education			0.0001	
Did not finish high school	78 (2.4%)	1 (0.4%)		0 (0%)
High school graduate	194 (6.0%)	8 (2.9%)		1 (7.1%)
Some college, did not graduate	183 (5.7%)	24 (8.6%)		2 (14.3%)
Associate degree	103 (3.2%)	20 (7.2%)		0 (0%)
Bachelor’s degree	491 (15.3%)	78 (28.0%)		3 (21.4%)
More than bachelor’s degree	622 (19.3%)	148 (53.1%)		2 (14.3%)
Master’s degree	-	97 (34.8%)		2 (14.3%)
Doctoral degree	-	51 (18.3%)		0 (0%)
Missing	1546 (48.1%)	0 (0%)		6 (42.9%)
Screening group choice			0.59	
Group 1 only	328 (10.2%)	27 (9.7%)		4 (28.6%)
Group 1 and 2	41 (1.3%)	6 (2.2%)		0 (0%)
Group 1 and T1D	38 (1.2%)	2 (0.7%)		0 (0%)
Group 1, Group 2, and T1D	2810 (87.4%)	244 (87.5%)		10 (71.4%)
Urbanicity			0.06	
Urban	2804 (87.2%)	256 (91.8%)		13 (92.9%)
Large rural city/town	306 (9.5%)	19 (6.8%)		1 (7.1%)
Small and isolated small rural town	107 (3.3%)	4 (1.4%)		0 (0.0%)
Preferred language at home			0.41	
English	3056 (95.0%)	267 (95.74%)		14 (100%)
Spanish	141 (4.4%)	9 (3.2%)		0 (0%)
Other	18 (0.6%)	3 (1.1%)		0 (0%)
Missing	2 (0.1%)	0 (0%)		0 (0%)
Consent portal language used			0.73	
English	3098 (96.3%)	273 (97.9%)		14 (100%)
Spanish	109 (3.4%)	6 (2.2%)		0 (0%)
Missing	10 (0.3%)	0 (0.0%)		0 (0%)
Health material help needed				
Never		219 (78.5%)		
Rarely		39 (14.0%)		
Sometimes		13 (4.7%)		
Often		6 (2.2%)		
Always		2 (0.7%)		
Device used to review information				
Smartphone		232 (83.2%)		
Laptop computer		102 (36.6%)		
Desktop computer		25 (9.0%)		
Tablet		12 (4.3%)		
I do not recall		2 (0.7%)		
Weeks from consent to survey				
Median (IQR)		20 (12.3-26.7)		
Min – Max		5.4–78.0		
Weeks from consent to interview				
Median (IQR)				25.9 (22.6-35.3)
Min – Max				16.4–39.6

The distribution of baby’s sex was generally similar across the overall enrolled cohort and survey respondents, with no statistically significant differences observed. Interview participants were more likely to have a female baby. Racial and ethnic representation varied somewhat. White mothers made up the majority in all groups, whereas Hispanic/Latino and Asian mothers were less represented in the survey and interview samples. Interview participants included a slightly higher proportion of African American/Black and Asian mothers compared to the overall parent cohort. The variation reflected a purposive sampling design aimed at increasing demographic diversity, as we intentionally recruited participants for interviews who were not white and provided consent to participate. Survey respondents tended to have higher levels of educational attainment compared to the overall parent cohort. Interview participants showed more varied educational backgrounds, with some holding advanced degrees and others reporting bachelor’s or professional degrees.

Screening group choices were generally similar between survey respondents and the broader parent cohort, with most opting to receive results from all three screening groups. Interview participants, however, were somewhat less likely to choose all groups and more likely to select only Panel one than the overall and survey samples. Of the 279 survey respondents, 246 received T1D results and therefore responded to T1D-specific survey items. Of the 14 interview participants, 10 received T1D results and discussed them in their interviews.

Urban residence was common across all *Early Check* participants, with slightly higher representation among survey respondents and interview participants. English was the predominant preferred language in all groups, with Spanish reported much less frequently and not at all among interviewees. Health literacy appeared to be high among survey respondents, with most reporting little to no difficulty reading medical materials. The majority accessed *Early Check* information using a smartphone, with smaller proportions using other devices such as laptops, desktops, or tablets.

### 3.2 Reasons for participating in *Early Check*

**FIGURE 1:**
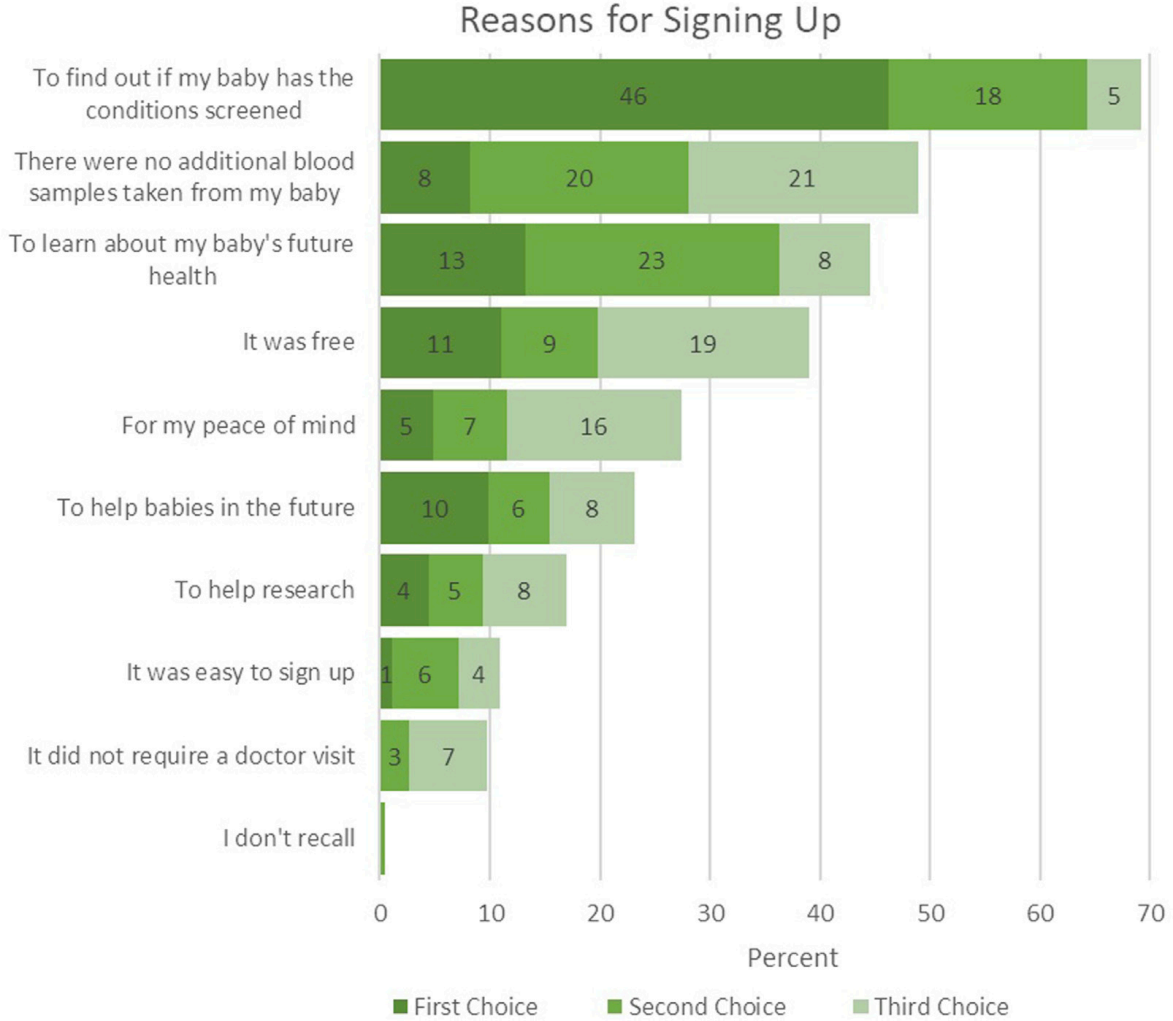
Reasons for enrolling in *Early Check* (n = 279).

Parents endorsed a range of reasons for enrolling their newborn in *Early Check* (Figure 1). The most common reason for enrolling was to find out if their baby has the conditions screened. Nearly half of participants selected this as their top reason, and over two-thirds included it among their top three choices. Nearly half were also motivated by the fact that no additional blood samples were needed, and a slightly smaller group enrolled to learn more about their baby’s future health. Cost and peace of mind were also meaningful factors. Altruistic reasons, such as helping future babies or supporting research, were less frequently mentioned but still notable, with about one in five participants endorsing them. Convenience was the least common motivator, with about one in ten participants influenced by the ease of enrollment or not needing a doctor’s visit.

Among the survey respondents who chose to screen their newborn for T1D risk, motivations leaned more heavily toward gaining health knowledge and preparing for the future (Table 2). The most common reason was to learn about their baby’s future health, with about two-thirds selecting this. Half were motivated by the fact that the program was free, and a notable portion appreciated that no additional blood samples were needed. Peace of mind and the ease of signing up were also frequently mentioned, though by a smaller share of participants.

**TABLE 2:** Reasons for selecting type 1 diabetes risk screening (n = 44).

Reasons	n	%
To learn about my baby’s future health	28	63.6%
It was free	22	50.0%
There were no additional blood samples taken from my baby	17	38.6%
For my peace of mind	14	31.8%
It was easy to sign up	9	20.5%
To help research	8	18.2%
To help babies in the future	7	15.9%
*Early Check* provides free additional testing for babies who get a ‘higher concern’ result for T1D	7	15.9%
Diabetes is part of my family’s medical history	7	15.9%
Type 1 diabetes only	0	0.0%^†^
Type 2 diabetes only	4	57.1%^†^
Both type 1 and type 2 diabetes	3	42.9%^†^
I know someone who has T1D	7	15.9%
It did not require a doctor visit	6	13.6%
I know someone who has T1D	1	2.3%
Another reason	0	0.0%
I do not recall	0	0.0%

^†^ Percentage based on respondents who reported a family history of diabetes (n = 7).

This question was asked only after 20 March 2025. The table includes only participants who answered “Yes” to signing up for type 1 diabetes risk screening (n = 44). Those who responded “Unsure” (n = 4) were excluded.

Qualitative interviews provided additional insights into these motivations. A majority of interviewees cited a desire to gain early knowledge that could support caregiving and decision-making. One parent explained, “Just so that way, if the results came back, something I would know now to be able to help guide our lifestyle choices … Just so I could be a better caregiver or parent, you know, to my baby” (PT123). Others saw the screening as a reassuring step toward preparedness: “We wanted to do whatever we could to, you know, increase knowledge and make sure that everything was fine” (PT831).

Several participants appreciated that the screening used the same blood already collected for other newborn tests, making participation more convenient. Additionally, some participants had a small amount of preexisting knowledge or family history related conditions, which heightened their anxiety. For instance, those with a family history of type 2 diabetes expressed increased concern about T1D screening results. As one participant explained: “Myself and her father are both diabetic and that really sparked my interest because I’m like, wow, I can see, you know, if my daughter is at risk for diabetes … I was 38 when I found out I was diabetic, Type 2, and it it’s hereditary. It runs in my family” (PT686). A few participants also mentioned enrolling because they wanted to contribute to research, seeing their participation to support broader scientific and medical advancements.

### 3.3 Perceptions and adequacy of information

#### 3.3.1 Perceived clarity and Sufficiency of educational material

**FIGURE 2:**
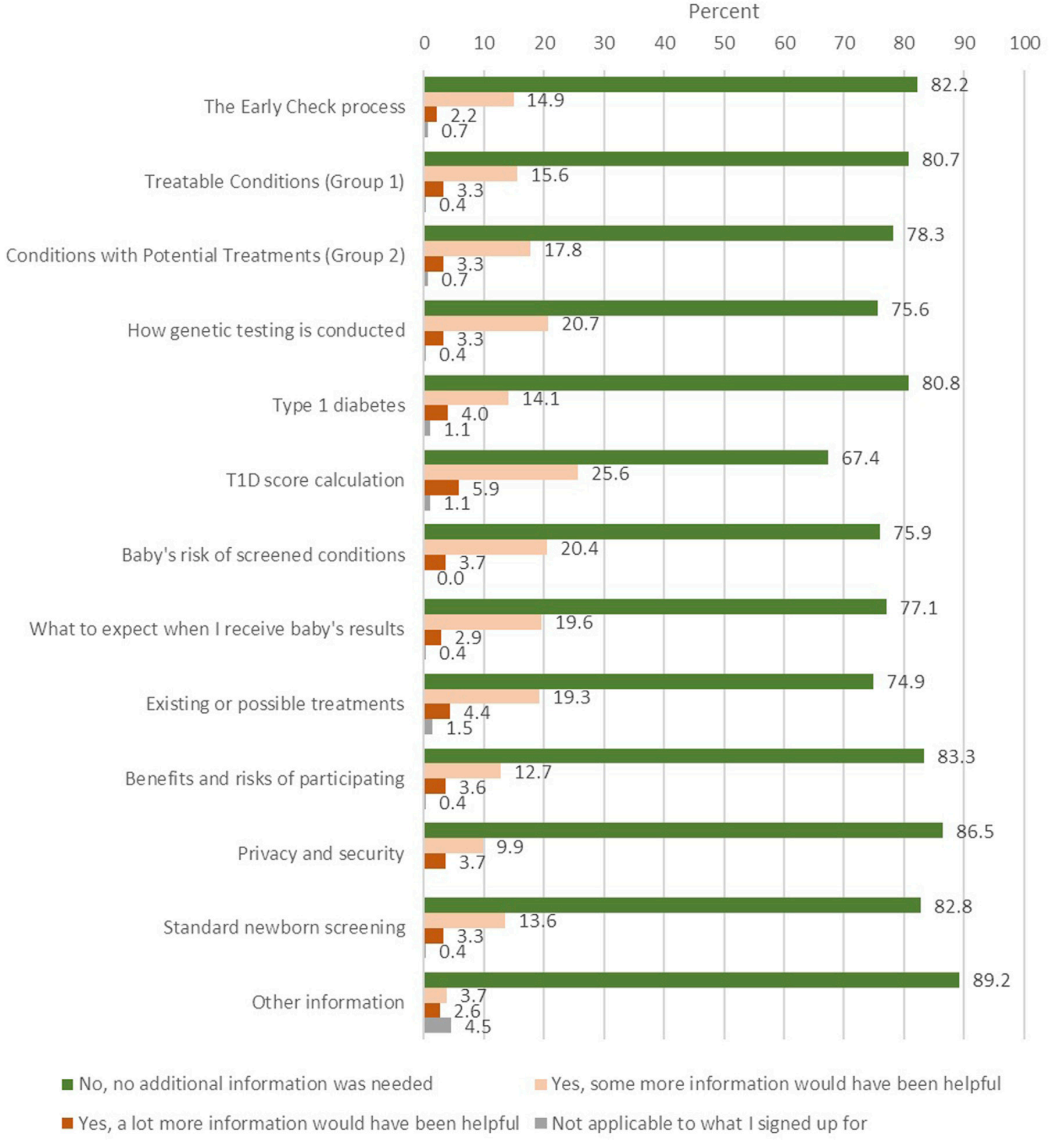
Information adequacy (n = 279).

Survey respondents generally felt well-informed, with a substantial majority (between 67% and 86% of respondents; *n* = 184 to *n* = 239) indicating that no additional information was needed across most topics (Figure 2). However, several areas emerged where a notable portion of respondents expressed a desire for more detail. The T1D score calculation topic generated the most requests for more information, with nearly one-third of respondents (31.5%) indicating that additional explanation would have been helpful. Almost a quarter of participants sought more detail on how genetic testing is conducted (24.0%), their baby’s risk of screened conditions (24.1%), and existing or possible treatments (23.6%). About one in five expressed interests in learning more about Treatable Conditions (Group 1; 18.9%), Conditions with Potential Treatments (Group 2; 20.4%), and what to expect when receiving their baby’s results (22.6%). The topic with the fewest requests for additional information was privacy and security (13.5%), suggesting that participants generally felt confident about how their data were protected. These findings suggest that although most participants felt adequately informed, a meaningful minority desired more clarity, particularly on complex or technical topics.

Interview data supported these trends. A majority of interviewees reported feeling that the information provided through the recruitment letter and *Early Check* portal was clear and sufficient. One parent reflected, “I felt like it was a good amount of information … I do not think there was anything I needed to get further information from anybody” (PT1316). Others appreciated the detail but still expressed general unease about the sensitivity of genetic data, as one participant explained: “I still felt really unsure … it’s another person’s life and their information … but there was so much information … I felt secure enough to do it” (PT123).

Overall, most participants expressed satisfaction with the educational content, but areas related to T1D and genetic testing stood out as requiring greater clarity. For example, one participant commented, “I wonder how the genetic risk score is calculated” (PT1439). Another elaborated that they would like to know, “the percent of children with this genetic alteration that still may not develop type 1 diabetes. But then again, that could be more confusing for people or parents. to kind of introduce more complexity than is necessary” (PT1551).

#### 3.3.2 Perceptions of the consent process and information clarity

**FIGURE 3:**
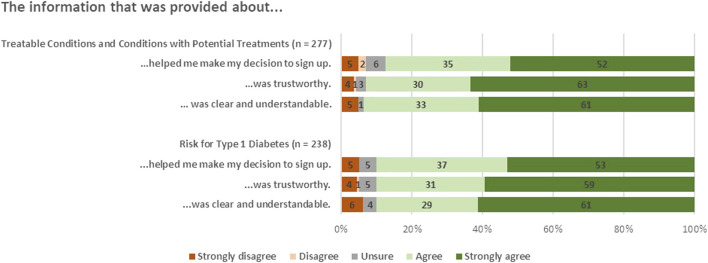
Reflections on information provided during enrollment.

Overall, survey respondents rated information in the *Early Check* portal highly for clarity, trustworthiness, and usefulness in guiding enrollment decisions (Figure 3). Ratings for T1D information were slightly higher in clarity and trustworthiness, and the perceived impact on decision-making was slightly lower compared to the information provided about monogenic conditions, both Treatable Conditions (Group 1) and Conditions with Potential Treatments (Group 2).

**FIGURE 4:**
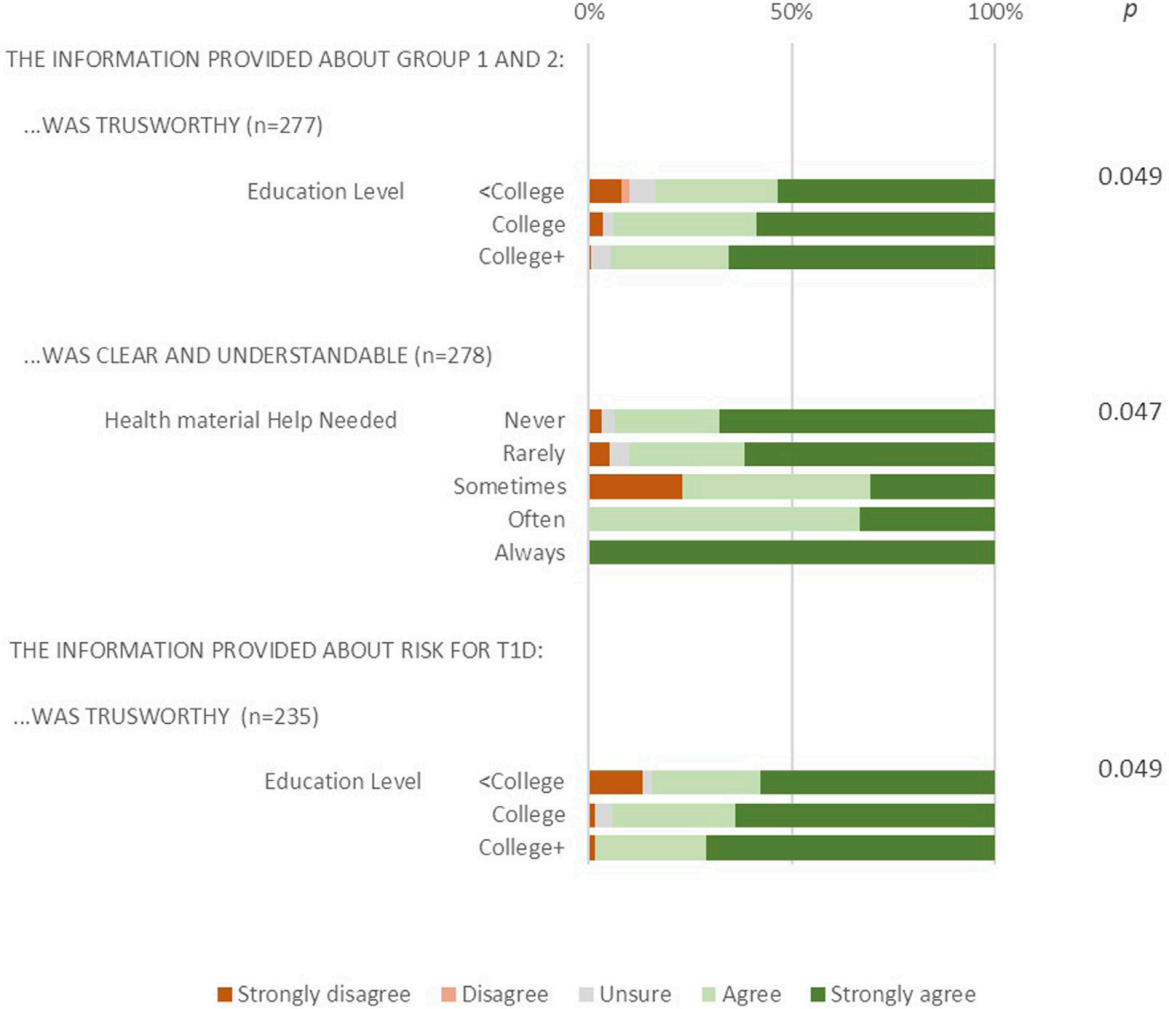
Assessment of trustworthiness and clarity of information subgroup analysis.

Assessments of the clarity, trustworthiness, and helpfulness of educational materials for both monogenic screening groups (Treatable Conditions and Conditions with Potential Treatments), as well as for T1D risk, were analyzed by education level, health literacy, and preferred language at home. Due to small sample sizes within some education categories and to improve the stability and reliability of statistical estimates, educational attainment levels were grouped into three categories: less than a bachelor’s degree, a bachelor’s degree, and more than a bachelor’s degree. Using Fisher’s Exact test, significant differences were observed based on education level. Participants with lower educational attainment levels were less likely to rate the information about both monogenic screening groups as well as T1D risk screening as trustworthy (*p* < 0.05; Figure 4). Health literacy was associated with perceptions of clarity; notably, participants with lower literacy tended to rate the materials as clear and understandable (*p* < 0.05; Figure 4).

Qualitative interviewees generally reported that the process was easy with no major concerns raised. However, one participant noted that the consent form was lengthy and required multiple reviews.

### 3.4 Participant knowledge and understanding of screening

**FIGURE 5:**
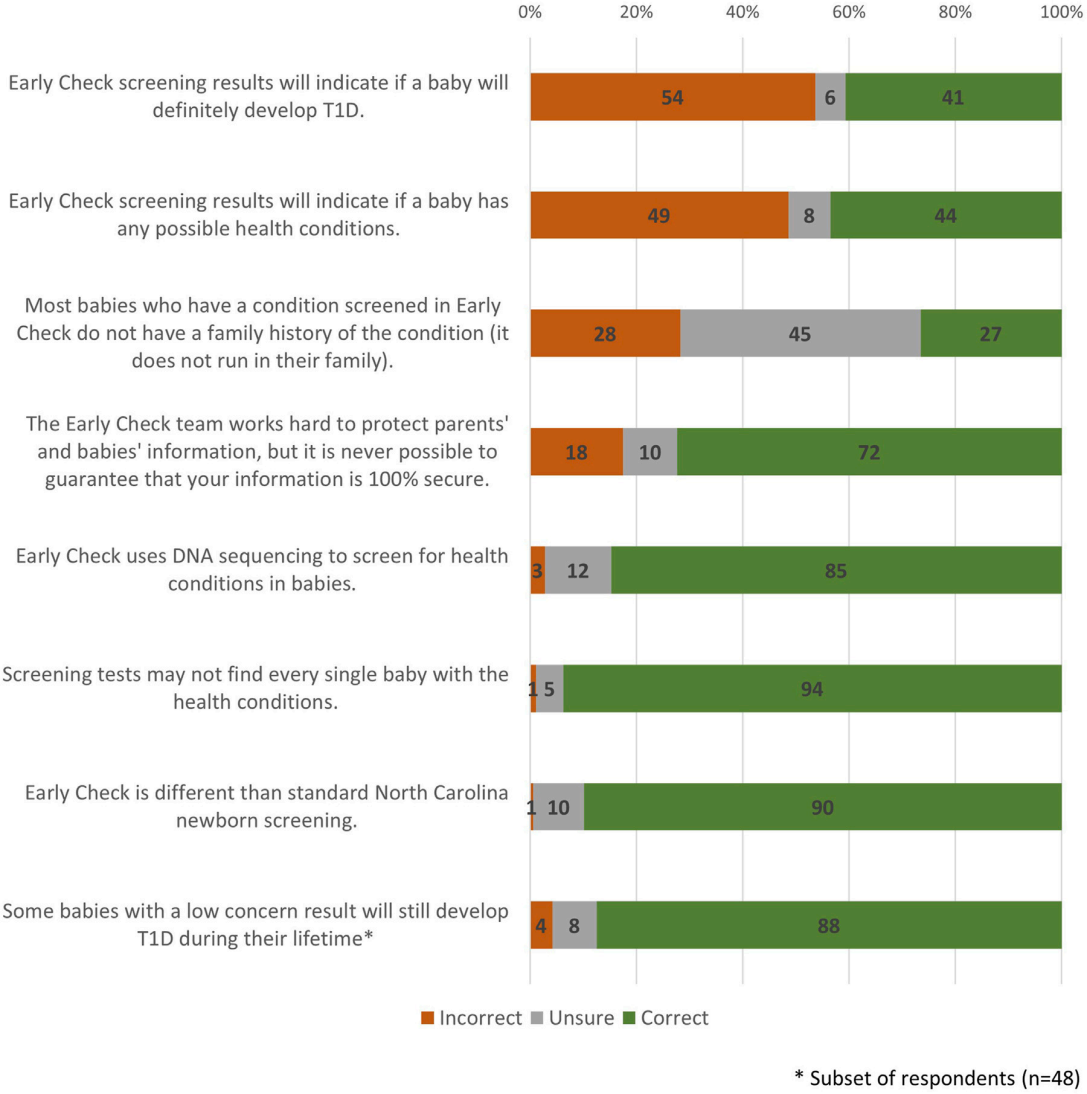
Participants knowledge of the *Early Check* study (n = 277).

The analysis revealed varying levels of understanding about *Early Check* screening. A notable portion of survey respondents held common misconceptions; for example, more than a third mistakenly believed that screening results could identify newborns who will definitely develop T1D (Figure 5). Nearly half incorrectly thought the screening could identify any possible health condition. Uncertainty about some knowledge items was also common, with about two in five unsure whether most babies with screened conditions have a family history. In contrast, some concepts were well understood by a strong majority. Most participants accurately recognized that *Early Check* uses DNA sequencing (85%), understood that it is distinct from standard North Carolina newborn screening (90%), and knew that not all affected babies may be detected through screening (94%).

Overall, about three out of five parents demonstrated an acceptable level of knowledge about *Early Check* screening (i.e., having a total score of five or higher; Table 3). In contrast, about two in five fell below this benchmark, suggesting that a sizable portion either had poor recall or may have benefit from clearer or more accessible information. On average, participants answered about two-thirds of the questions correctly. An exploratory question added to later surveys (n = 48) found nearly 9 out of 10 participants correctly understood that some babies who receive a low concern result may still go on to develop T1D.

**TABLE 3:** Components of informed choice among participants.

Measure	Description	Items (range)	Mean (SD)	Median (IQR)	Outcome
MMIC knowledge scale (n = 276)	Accurate and sufficient understanding of key concepts	Seven True/False/Unsure questions (0-7)	69.1% (17.5%)	5.0 (4-6)	Acceptable (≥5): 60.9% Less than acceptable (<5): 39.1%
MMIC attitude scale (n = 271)	Attitude towards participating in EC	Five 5-point Likert items (0-20)	1.2 (2.6)	0 (0-1)	Positive (0-6): 94.8% Neutral (7-13): 4.4% Negative (14-20): 0.7%

A logistic regression analysis explored whether factors such as education level, health literacy, screening group selection, and language spoken at home were linked to having adequate knowledge about *Early Check* screening. Of these, only education level showed a significant association with having adequate knowledge based on our threshold of five or more questions correct out of seven. Participants with less than a college degree were significantly less likely to demonstrate adequate knowledge compared to those with more than a college degree (OR = 0.42, 95% CI = 0.22-0.88, *p* < 0.01). Participants with a college degree were somewhat less likely to achieve adequate knowledge status than those with a higher degree (OR = 0.8, CI = 0.45, 1.42), this difference was not statistically significant (*p* = 0.44).

### 3.5 Attitudes towards participation

Survey respondents’ attitudes toward participation were generally very positive, with the vast majority expressing a favorable view (see Table 3). Participants who selected screening for T1D risk reported more favorable attitudes compared to those who did not (96.9% vs. 83.3% respectively, p = 0.001), although the number of respondents who did not select T1D risk screening was relatively small in our sample.

### 3.6 Experience receiving and understanding results

#### 3.6.1 Actions taken after receiving screening results

Parents received up to two notification emails when their baby’s results were ready. Nearly all survey respondents reported reading the notification email and viewing the results in the *Early Check* portal, indicating a high level of initial engagement (see Table 4). For the monogenic results (i.e., Treatable Conditions [Group 1] and Conditions with Potential Treatments [Group 2]) the majority of participants read both the email notification and the summary results on the *Early Check* portal. A smaller, but still substantial, number reviewed the detailed results report provided by the commercial sequencing laboratory. Fewer respondents downloaded the monogenic or T1D reports, with download rates somewhat lower overall but relatively consistent between the two groups, with just under two-thirds in each case.

**TABLE 4:** Engagement with and sharing of screening results.

Engagement	Monogenic results	T1D results
Action Taken		
Read results ready email	268/278 (96.4%)	216/228 (94.7%)
Viewed summary result on *Early Check* portal (for T1D the entire laboratory report was embedded into the results portal page)	263/268 (98.1%)	209/215 (97.2%)
Read laboratory report	221/260 (85.0%)	n/a
Downloaded report^†^	132/215 (61.4%)	121/206 (58.7%)
Who have you told about your baby’s results (check all that applies)		
The baby’s father	218/263 (82.9%)	168/209 (80.4%)
Other relative(s) of the baby	82/263 (31.2%)	58/209 (27.8%)
Friends	25/263 (9.5%)	21/209 (10.1%)
My baby’s healthcare provider	34/263 (12.9%)	23/209 (11.0%)
My healthcare provider	8/263 (3.0%)	7/209 (3.4%)
Someone else	4/263 (1.5%)	3/209 (1.4%)
No one	26/263 (9.9%)	30/209 (14.4%)
Do you plan to tell anyone else about your baby’s results?		
No	114/261 (43.7%)	98/207 (47.3%)
I do not know or unsure	93/261 (35.6%)	68/207 (32.9%)
Yes	54/261 (20.7%)	41/207 (19.8%)
Who do you plan to tell?		
The baby’s father	14/54 (25.9%)	14/41 (34.2%)
Other relative(s) of the baby	22/54 (40.7%)	18/41 (43.9%)
Friend(s)	15/54 (27.8%)	13/41 (31.7%)
My baby’s healthcare provider	23/54 (42.6%)	22/41 (53.7%)
My healthcare provider	7/54 (13.0%)	6/41 (14.6%)
Someone else	1/54 (1.9%)	0/41 (0.0%)
I Watched one of the educational videos about T1D or my baby’s result		
Yes	n/a	47/169 (27.8%)
No	n/a	122/169 (56.5%)
The educational videos were helpful for understanding my baby’s risk for T1D		
Strongly disagree	n/a	1/47 (2.1%)
Disagree	n/a	0/47 (0.0%)
Unsure	n/a	4/47 (8.5%)
Agree	n/a	23/47 (48.9%)
Strongly agree	n/a	19/47 (40.4%)

^†^ Laboratory Report for monogenic results and Research Report for T1D results.

#### 3.6.2 Sharing of results with others

Most parents shared their baby’s screening results with at least one other person, most often with the baby’s father (Table 4). About one in ten parents shared the results with their baby’s healthcare provider.

#### 3.6.3 Usefulness and uptake of T1D educational videos

Among survey respondents who elected T1D risk screening and viewed their results on the *Early Check* portal, engagement with the educational videos about T1D was relatively limited. Fewer than one in three reported watching at least one video, and the majority did not engage with the videos at all (Table 4). However, among those who did watch them, nearly all found them helpful in understanding their baby’s risk for T1D.

#### 3.6.4 Self-reported understanding of screening results

Among participants who reported having viewed the summary report on the *Early Check* portal, most reported a strong understanding of their baby’s screening results (Table 5). For both monogenic and T1D risk screening, a large majority–about three out of four or more–said they understood their results “quite or bit” or “extremely well.” Only a minority of participants reported limited understanding.

**TABLE 5:** Self-reported understanding of baby’s results.

	Monogenic results (n = 260)	T1D results (n = 208)
How well do you understand your baby’s results		
Not at all	4 (1.5%)	2 (1.0%)
A little bit	12 (4.6%)	7 (3.4%)
Moderately well	40 (15.4%)	36 (17.3%)
Quite a bit	68 (26.2%)	50 (24.0%)
Extremely well	136 (52.3%)	113 (54.3%)

Survey respondents’ understanding of their baby’s screening results varied by education level, health literacy, and language spoken at home (Table 6). Among those reviewing their baby’s monogenic results, understanding increased slightly with higher education, but this difference was not statistically significant. However, for those reviewing T1D risk results, education level was significantly associated with higher self-reported understanding (*p* = 0.03). Tukey pairwise comparisons revealed that participants with a college degree or more advanced education reported significantly higher understanding than those with less than a college degree (*p* < 0.0001).

**TABLE 6:** Median Self-Reported Understanding Rating^1^ of Results by subgroup.

Subgroup	Monogenic results				T1D results			
	n	Average	Test statistic	*p*-value	n	Average	Test statistic	*p*-value
Highest degree		Mean (SD)	*F* (2) = 1.1	0.32		Mean (SD)	*F* (2) = 3.6	0.03
< College	42	3.0 (1.1)			33	3 (2-4)		
College	74	3.3 (1.0)			63	4 (3-4)		
College +	144	3.3 (1.0)			112	4 (3-4)		
Health material help needed		Median (IQR)	*H* (4) = 16.8	0.002		Median (IQR)	*H* (4) = 18.02	0.001
Never	208	4 (3-4)			165	4 (3-4)		
Rarely	33	3 (2-4)			27	3 (2-4)		
Sometimes	12	3 (2-4)			9	3 (2-3)		
Often	6	2 (1-3)			5	2 (2-3)		
Always	1	4 (4-4)			2	4 (4, 4)		
Language		Median (IQR)	*H* (2) = 7.9	0.02		Median (IQR)	*H* (2) = 9.22	0.01
English	249	4 (3-4)			199	4 (3-4)		
Spanish	8	2.5 (1.5-3.5)			6	2.5 (1-3)		
Other	3	2 (2-3)			3	2 (2-3)		
Portal language			*H* (1) = 4.2	0.04			*H* (1) = 2.5	0.11
English	255	4 (3-4)			205	4 (3-4)		
Spanish	5	2 (1-3)			3	1 (1-4)		

^1^ How well do you understand your baby’s result?: 0 = Not at all, 1 = A little bit, 2 = Moderately well, 3 = Quite a bit, 4 = Extremely well.

H: Kruskal–Wallis Test.

F: ANOVA.

Health literacy was also significantly related to comprehension in both result types, with lower comprehension observed among participants who more frequently needed help reading health materials (monogenic results: *p* = 0.002; T1D risk results: *p* = 0.001). Respondents who never needed help reported the highest comprehension (median = 4, “extremely well”), and those who needed help “sometimes” or “often” reported lower comprehension with median scores ranging from 2 to 3.

Language preference showed a similar pattern: English speakers had the highest self-reported understanding for both result types, whereas participants who preferred Spanish or another language reported substantially lower levels of self-reported understanding, generally rated around “moderately well.” These differences were statistically significant for both monogenic results (*p* = 0.03) and T1D risk results (*p* = 0.01). To assess whether a similar pattern would emerge based on the language used during the consent process itself, we tested differences in self-reported understanding using the language portal used during consent (English or Spanish). This analysis revealed a comparable trend: participants who used the English-language portal reported higher understanding for both monogenic and T1D risk results, while those who used the Spanish-language portal reported lower understanding. The difference based on portal language was statistically significant for monogenic results (*p* = 0.04) but not for T1D risk results (*p* = 0.11). Notably, there were fewer participants who completed the consent process in Spanish than those who reported Spanish as their preferred language, which likely limited the statistical power of the portal language analysis–particularly for T1D risk results.

Qualitative interviews supported these findings and provided additional insight into how parents interpreted their results. Several interviewees described feeling anxious before opening the results but were reassured upon seeing a “low concern” or “negative” result clearly marked at the top of the report. One parent noted about the monogenic results report, “Right at the top it says result negative. That way you do not have to kind of go searching to figure it out while you’re a little bit nervous” (PT1551). Another added about the T1D results report, “Low concern is just right there and it's highlighted … that was really helpful” (PT554).

Participants consistently emphasized that visual elements were critical for understanding. “The graphics make it super easy to understand,” said one parent (PT130). The inclusion of color-coded bars, percentiles, and infographics helped participants interpret risk levels and place their child’s results in context. This feedback was specific to the T1D results; monogenic findings were returned using a standard laboratory report without added visual enhancements. Several parents also appreciated the inclusion of symptom lists and action-oriented messaging. One stated, “The list of potential symptoms … it was good to know things to look out for in the future” (PT831). Another reflected, “The concern level and kind of what your action item is was helpful … like do not just brush symptoms under the rug” (PT1551).

At the same time, a few participants noted that some of the language or genetic details, particularly in the monogenic results report, might be challenging for those without a medical background. “I thought that level of detail was great … but I do not know if that would be more confusing for someone who’s not a scientist” (PT1551). One participant suggested adding contextual analogies for the T1D risk results: “When you say like 2% risk … maybe you can say, out of 10,000 people, how many would actually develop it” (PT1927).

### 3.7 Decision regret

A vast majority of participants expressed no regret about their decision to participate in *Early Check* (Table 7). One in four experienced mild regret, and only a small portion (6.3%) reported high levels of regret. In a test for differences among groups, there were no differences by urbanicity, education level, or mother’s age (Table 7).

**TABLE 7:** Decision regret overall and by subgroup.

Subgroup	n	Decision regret			*p-*value
		No regret	Mild regret	High regret	
Overall	271	68.6%	25.1%	6.3%	
Education					0.02
< College	49	29 (59.2%)	13 (26.5%)	7 (14.3%)	
College	78	48 (61.5%)	25 (32.1%)	5 (6.4%)	
College +	144	109 (75.7%)	30 (20.8%)	5 (3.5%)	
Race					0.10
African American/Black	16	9 (56.3%)	3 (18.8%)	4 (25.0%)	
Asian	9	5 (55.6%)	2 (22.2%)	2 (22.2%)	
Hispanic/Latino or Spanish	13	8 (61.5%)	4 (30.8%)	1 (7.7%)	
White	205	145 (70.7%)	51 (24.9%)	9 (4.4%)	
Two or more race	24	15 (62.5%)	8 (33.3%)	1 (4.2%)	
Unknown	4	4 (100.0%)	0 (0.0%)	0 (0.0%)	
Selected T1D risk screening					0.006
Yes	239	170 (71.1%)	58 (24.3%)	11 (4.6%)	
No	32	16 (50.0%)	10 (31.3%)	6 (18.8%)	

*p-*values calculated using Fisher’s Exact test due to small cell sizes.

Decision regret varied based on education level and T1D screening participation, but not by race or ethnicity (see Table 7). Participants with higher levels of education were significantly more likely to report no regret and less likely to report high regret. Similarly, those who opted into T1D screening reported lower regret overall compared to those who did not participate. Although differences by race were not statistically significant, there were some patterns indicating that participants identifying as African American/Black or Asian reported higher levels of regret (25.0% and 22.2% respectively) compared to White participants (4.4%).

Even participants who misunderstood the relationship between their child’s results and family history, such as believing type 2 diabetes increased their child’s risk for T1D, still expressed satisfaction with their decision-making.

Interview participants reinforced these findings. All expressed satisfaction with their newborn’s participation and said they would be willing to engage again. Most said they would recommend participation to others, although a few noted they would consider how their friends might respond before doing so.

These survey findings were echoed in the qualitative interviews when participants were asked whether they would make the same decision again, with participants consistently describing the experience as reassuring, easy, and worthwhile, even when no actionable results were returned. One parent reflected, “I felt confidence in the whole process … it was what I expected … and I am happy to have the information” (PT1176). Another parent added, “If it was offered to me again for a second child, I would absolutely do it again” (PT890), and another noted, “I just wish we had had that for [our older son] too … to have that more information and peace of mind” (PT1752).

Several participants emphasized how the process brought peace of mind and relieved postpartum anxiety (PPA). One parent shared, “It gave me a good peace of mind … I had really bad PPA, especially the first 12 weeks … it actually was like, my postpartum … fear that she’s gonna be sick kind of dissipated when we got the results” (PT554).

Despite minor misunderstandings, such as confusing Type 1 and Type 2 diabetes or overestimating the predictive value of results, participants still described their decision as positive and empowering. “Even knowing she did not have an issue, we would choose to do it again … because we would want to know regardless” (PT831).

Importantly, participants also strongly endorsed the program to others. “I definitely have recommended this program to both of my cousins,” said one parent. “Why not? It’s offered right there, the child does not have to get restuck … do it for them” (PT1391).

This sense of reassurance was particularly strong among participants who expressed pre-existing health concerns or had family histories of conditions. As one participant put it: “It was effortless … I can get the reassurance I like. I do not need to keep worrying about genetic disease or keep looking for signs and symptoms” (PT1439). Another emphasized the program’s value even without a clinical result: “You’re contributing to research … and it’s helpful for all the new parents to know, to have more information about their newborns” (PT1927).

## 4 Discussion

Our mixed-methods evaluation of the *Early Check* model provides valuable insights into the feasibility, acceptability, and effectiveness of electronic-based education, consent, and return of results processes in large-scale gNBS. Overall, parents responded favorably to participation and demonstrated generally adequate understanding, yet topic-specific gaps persisted–particularly around what genomic screening can and cannot reveal, with more technical or conceptually complex topics such as T1D risk scoring and genetic testing processes requiring clearer explanations or supplemental resources. This overall pattern is consistent with earlier electronic-consent evaluations showing high acceptability, trust, and limited regret (Peay et al., 2022), with contextual factors–such as sequencing-focused content and emphasis on screening limitations–likely shaping the observed variability. These results align with findings from the CSER consortium, which reported high parental satisfaction and low decision regret in genomic sequencing studies despite varying levels of understanding (Gray et al., 2014). Similarly, the BabySeq Project observed that parents generally experienced low decisional regret and comprehension of complex genomic information varied (Pereira et al., 2021). Armstrong et al. (2022) further reported that although parents supported newborn genomic sequencing, they expressed stronger support for standard newborn screening. The GUARDIAN study further supports these findings, highlighting high parental satisfaction alongside challenges in ensuring comprehensive understanding of genomic results in diverse populations (Ziegler et al., 2025).

Beyond topic-specific gaps, variations in perceived clarity and understanding also appeared to relate to parents’ health literacy levels, despite health literacy not being associated with quiz-based knowledge scores, offering further insight into how parents with different literacy levels engaged with the *Early Check* materials. Parents with lower health literacy were more likely to rate the educational materials for both Treatable Conditions (Group 1) and Conditions with Potential Treatments (Group 2) as clear and understandable. This result was unexpected given prior evidence that lower literacy is typically associated with reduced comprehension of health information: a recent review of health literacy and laboratory result communication highlighted that limited literacy was among the most salient barriers to understanding medical information (Lazaro, 2023). In contrast, the layered and simplified design of the *Early Check* materials may have enhanced perceived clarity across literacy levels. However, perceived clarity does not necessarily indicate full comprehension; in a 2024 scoping review of genomics education research, self-reported confidence and perceived understanding often exceeded demonstrated knowledge (Nisselle et al., 2024). It is also possible that some participants with lower literacy perceived the materials as clear due to limited awareness of their own comprehension gaps, a distinction observed between self-reported and performance-based literacy measures (Song, 2025). Together, these findings underscore the importance of evaluating both perceived and objective comprehension to ensure equitable understanding of complex genomic concepts. Future research should explore how to best assess and bridge gaps between perceived clarity and actual understanding across literacy levels, particularly in the context of digital, self-directed genomic education materials.

Motivations for participation were largely shaped by a desire for immediate health knowledge and the convenience of a low-burden, no cost program. These motivations are similar to those expressed by birthing parents of our prior, non-sequencing based *Early Check* study in which participants were also motivated by ease of use, accessibility of a self-directed web-based portal, and the opportunity to receive individual research result (Cope et al., 2023). Parents who elected to participate in T1D risk screening placed particular value on understanding their child’s future health and appreciated the availability of additional free testing. Although a family history of diabetes was less frequently endorsed overall, when it was cited, it was most commonly associated with type 2 diabetes or a combination of type 1 and type 2 diabetes. This likely represents a common misconception that type 1 and type 2 diabetes may have a similar etiology. These findings align with the literature on the utility of genetic testing from the parental perspective, which highlights that parents derive not only clinical utility but also personal utility—such as cognitive (valuing the opportunity to learn more about their child’s health), emotional (peace of mind), behavioral (planning), and social (altruism or supporting research) benefits—from participation in gNBS programs (Hayeems et al., 2021).

Overall knowledge and comprehension of gNBS were generally acceptable but varied substantially by specific content areas. Respondents demonstrated strong self-reported understanding of basic screening concepts consistent with previous studies (Peay et al., 2022). On items that tested their knowledge of key concepts, most scored well on specific items, such as understanding that not all affected children will be detected and that a low concern result does not rule out T1D risk. The high self-rating of understanding and overall high knowledge score may be in part due to a thoughtful consent and education strategy that deliberately incorporated layered information, tailoring, and multimedia tools based on formative research and best practices.

Despite these efforts, participants’ understanding of key concepts–such as whether *Early Check* screening results can determine if a baby will definitely develop T1D or reveal any possible condition–remained imperfect, raising important questions about the limits of even well-designed educational efforts. Areas of misconceptions included the implications of T1D screening results, reflecting broader challenges in interpreting risk scores–especially for multifactorial conditions like T1D, where elevated risk does not imply certainty of diagnosis (Sun et al., 2025). It is important to note that some knowledge questions may have been difficult to interpret, particularly for parents without prior familiarity with genomic concepts (Schmidt et al., 2022), which could have affected response accuracy. Our analysis found that education level was a significant factor of having adequate knowledge. This finding reinforces longstanding concerns about health literacy and equity in genomic education, suggesting that individuals with lower educational attainment may need additional support to fully understand complex screening information.

These gaps in knowledge may stem not only from the complexity of communicating probabilistic and genetic risk, but also from limitations in recall, as parents completed follow-up surveys several weeks to months after enrollment, meaning that even well-informed participants may not have retained all details. Therefore, interpretation of knowledge scores should be approached with caution given the potential for recall bias, which also highlights the broader challenge of retaining information–underscoring the need for designing educational content that is not only accessible at the time of consent but also promotes sustained comprehension over time.

Parental engagement and understanding of negative/normal results are critical in gNBS programs, particularly given the practical limitations of providing one-on-one genetic counseling to every family, including those receiving negative results. Although most parents accessed and reviewed the lay summary promptly, fewer engaged with the more detailed reports or educational videos. This limited engagement may reflect satisfaction with summary results, especially for those receiving negative results. Another interpretation for the limited engagement with detailed materials may be indication of misalignment between the content format and needs or preferences. To better interpret these engagement patterns, future iterations of *Early Check* could incorporate more user testing to understand how parents interact with different types of information and to determine whether adjustments in format, content, or delivery are needed to support robust engagement and informed understanding across diverse user groups (Sørensen et al., 2012).

Patterns of parental engagement with negative or normal screening results highlight the complex balance between reassurance, perceived relevance, and information needs in gNBS programs. The generally prompt access to results suggests strong initial interest, yet limited engagement with more detailed materials point to possible saturation once parents felt reassured that their child’s results were normal. Differences in engagement between monogenic and T1D risk information may further reflect how parents assess the personal relevance or actionability of results–echoing broader challenges in sustaining attention to genomic risk information that carries limited immediate consequence (Goldenberg et al., 2013). These observations underscore that, in digital models like *Early Check*, high accessibility alone does not guarantee deep engagement or comprehension.

Although most parents, particularly those who received T1D risk information, felt confident in their understanding of their baby’s results, a small subset continued to experience uncertainty or limited comprehension. Self-reported comprehension of results was consistently higher among participants with higher education, better health literacy, and a preference for English, with notable gaps in understanding among those with lower education attainment and non-English language preference. These findings underscore the need for educational strategies tailored to varying literacy levels and cultural backgrounds, particularly when communicating results from complex genomic risk information (Connolly et al., 2023). Despite our efforts to address these issues–including translating all content and materials into Spanish, offering Spanish speaking support staff to answer questions, conducting focus groups during the design phase to incorporate perspectives from diverse backgrounds, and ensuring materials were accessible at lower literacy levels–we still observed disparities in knowledge and understanding. This highlights the persistent and multifaceted nature of these challenges and underscore the need for further research to develop more effective, inclusive communication strategies.

Despite some knowledge gaps, most respondents expressed positive attitudes toward *Early Check*, perceived burden to be low, and had low levels of regret. These findings indicate that positive attitude towards participation can coexist with incomplete understanding when respondents feel reassured, trust the program, and experience a low burden of participation (O'Doherty et al., 2024). Levels of regret were similar across different races although higher education and participation in T1D screening separately were associated with lower levels of decision regret. However, it is of note that a quarter of African American/Black survey respondents reported “High Regret” regarding participation. This finding did not emerge in the qualitative interviews; however, we did not purposively sample individuals based on regret scores, and it is possible that those who felt regret may have been less willing to participate in follow-up interviews, introducing potential bias in our understanding of their perspectives. These findings suggest that, although a noteworthy quarter of African American/Black respondents reported high regret, race itself was not a significant predictor of decision regret; rather, perceived clarity and confidence during decision-making may play a more important role in reducing feelings of conflict or remorse after the decision is made (Altin and Stock, 2016; Burton, Blundell, Jones, Fraser & Elwyn, 2010). However, this interpretation is based primarily on qualitative data from a relatively small and non-representative subset of participants and should therefore be viewed as exploratory. Future research should further explore how race, clarity, and confidence interact to shape experiences of decision regret.

A key strength of the *Early Check* electronic consent model is its scalability, leveraging existing newborn screening infrastructure and electronic consent methods to reach a broad and geographically dispersed population. Electronic recruitment strategies, such as invitations through patient portals, have also shown promise for reaching expectant and new parents efficiently (Halpin et al., 2025). It is worth noting that the survey and interview participants differed demographically from the broader screen-negative cohort–most notably with greater representation of White, high educated, urban, and English-speaking individuals–which may influence how broadly the findings apply. This demographic skew may help contextualize the broadly positive attitudes and low regret observed, as these groups may experience greater trust in research and public health programs. Such differences highlight the pervasive and deep challenge facing researchers and public health: namely, how to achieve gNBS at scale while ensuring equity in follow-up care and trust in public health programs despite persistent gaps in understanding complex concepts related to probabilistic and genomic risk, limited access to personalized support, and underrepresentation of diverse populations in formative research (Khoury et al., 2022). Without deliberate attention to cultural competence, health literacy, and digital access, such programs may inadvertently widen existing healthcare disparities, particularly among historically underserved populations, and perpetuate the lack of genomic data needed to better understand how genes affect health in people from non-European ancestries.

Although our findings align with earlier research, it is important to consider the program’s distinct design when drawing comparisons. Unlike most other studies, *Early Check* used a fully remote digital consent process, carefully developed in both English and Spanish to support accessibility and inclusion. The *Early Check* platform was developed through a multidisciplinary collaboration integrating expertise in newborn screening, clinical genetics, public health, ethics, and health communication (Bailey et al., 2019). This collaborative approach facilitated the integration of best practices across content development, digital infrastructure, and clinical research protocols (Beskow et al., 2017; Corneli et al., 2024; Gesualdo et al., 2021; Kaur et al., 2025). Extensive formative research, including focus groups and user testing of outreach and consent material informed the design and communication strategies and the development of user-centered electronic permission portal. Results were returned through a secure electronic portal that delivered information in simplified, lay-friendly language, accompanied by videos and educational resources in both languages. This digital model may enhance accessibility and support understanding, particularly for families who prefer flexible, self-paced formats or face logistical barriers to in-person participation. For example, parents can revisit the materials as needed or share them with other caregivers, which may help reinforce comprehension and reduce decisional regret.

While the primarily digital design of *Early Check* offers notable advantages in scalability, efficiency, and convenience, it also presents certain limitations. Chief among these is the absence of direct, in-person interaction may limit opportunities for immediate clarification or personalized support–factors that could be particularly important for families with lower health literacy or heightened concern. Although digital tools increase scalability and convenience, they may not fully replicate the reassurance and dialogue that in-person counselling can offer. These factors are important to consider when evaluating parental experiences with *Early Check* and in planning for future models of gNBS.

Our findings suggest several areas for improvement. Some participants reported feeling overwhelmed by the volume of information, suggesting that additional streamlining of consent materials may help reduce perceived burden and enhance accessibility. Knowledge assessment data revealed frequent misunderstandings about the probabilistic nature of risk scores as well as the limited scope of screening panels. Additionally, confusion between T1D and T2D suggested a need for clearer communication about how genetic risk relates specifically to T1D. Future revisions to educational content should emphasize what a GRS does and does not indicate, clarify the limited range of conditions screened, and explicitly distinguish between disease types to avoid conflation. These efforts must balance the goal of streamlining and tailoring consent and reporting content with the obligation to ensure informed choice and adequate understanding of results.

### 4.1 Limitations

Despite the strengths of this mixed-methods design, some limitations should be acknowledged. First, survey and interview participants were self-selected and more likely to be White, highly educated, and urban-dwelling when compared with the overall sample of participants in *Early Check* who had a negative screening result, which may limit generalizability to more diverse populations. Second, although we included both quantitative and qualitative data, our sample size for interviews was modest and may not reflect the full range of parent experiences. In addition, this evaluation included only parents who received normal (negative) screening results and who chose to participate in *Early Check*. Parents whose infants received positive or uncertain results, as well as those who declined participation, were not represented here as they will be evaluated and reported separately. These groups may have different levels of understanding, decision satisfaction, or trust, which could influence findings and further limit representativeness. Additionally, due to the opt-in, electronic nature of the study, individuals with limited digital literacy or internet access may have been underrepresented in *Early Check*. Another limitation for consideration is the use of established scoring methods based on the MMIC framework which applies equal scores for all knowledge items. Some items may have greater implications for parents’ decisions and understanding than others. Developing a weighted or impact-adjusted version of the measure could provide a more nuanced insight into informed decision-making in future work. Lastly, variation in time since consent, result receipt, and survey or interview participation likely influenced recall, engagement, and reported experiences.

## 5 Conclusion

As gNBS becomes increasingly integrated into personalized medicine, models like *Early Check* provide valuable lessons for balancing accessibility, participant understanding, and ethical engagement. Our findings highlight that electronic consent and education platforms can successfully support informed participation and generally positive experiences with the vast majority (i.e., those with normal/screened negative results). It is important to note that *Early Check* implemented these strategies based on a strong empirical foundation, extensive community engagement, and substantial investment in education and consent infrastructure. Yet, despite substantial investments to achieve these advantages, important gaps remain–particularly among those with lower health literacy–raising important questions about the true scalability of efforts to “reach everyone” through digital education and consent in large-scale public health genomics. These gaps underscore the need for continued research, innovation, and investment in communication strategies that meet families where they are. Researchers should continue to refine consent and education approaches to better support diverse populations, particularly those historically underrepresented in genomic research. In addition, policymakers, healthcare systems, and research institutions should work collaboratively to develop scalable, equitable, and ethically sound gNBS programs that empower families with actionable information while safeguarding against potential misunderstandings and inequities. Ultimately, a focus on participant-centered design, informed choice, and ongoing evaluation will be critical to ensuring that the expansion of gNBS fulfils its promise of improving health outcomes for all newborns, regardless of their background or circumstances.

## Data Availability

The datasets presented in this article are not readily available because The raw qualitative interview and survey data underlying this article are not publicly available due to participant confidentiality and ethical restrictions, but requests for access can be directed to the corresponding author. Requests to access the datasets should be directed to agwaltney@rti.org.
