# Supplementary figures and images for: Perspectives of parents receiving normal results from genomic newborn screening: a mixed-methods evaluation from the early check program
Source: Front Genet. 2025 Dec 3;16:1704364. doi: 10.3389/fgene.2025.1704364 (PMC12709501; doi:10.3389/fgene.2025.1704364)

**
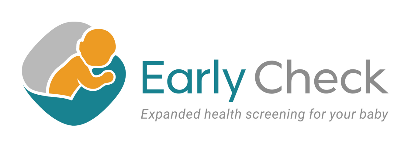
Supplementary File 2. Interview Guide for Screened Negative Participants**


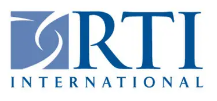


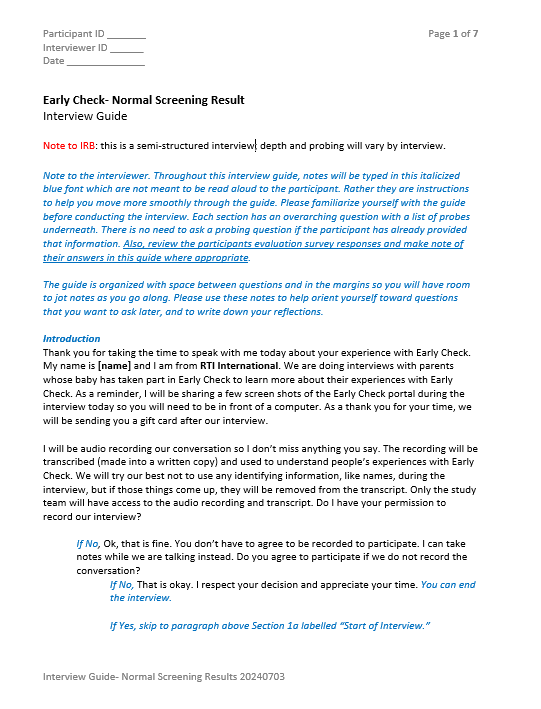


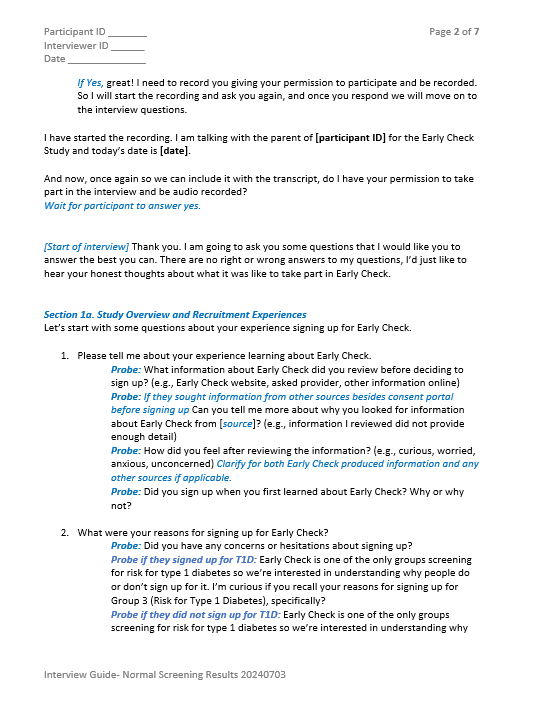

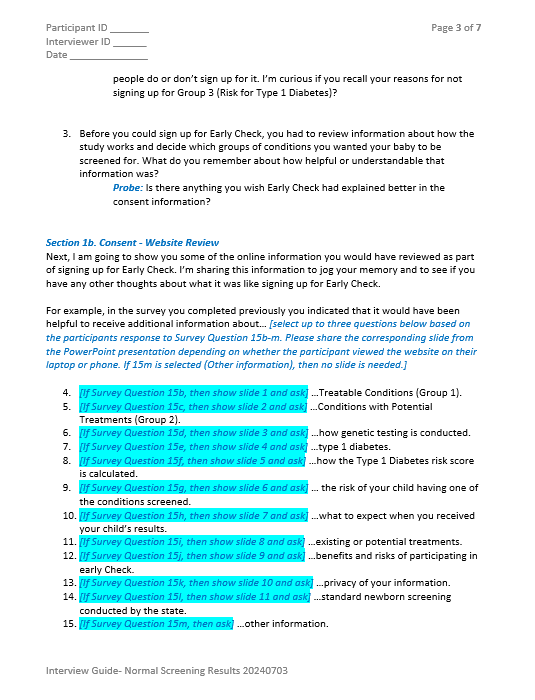

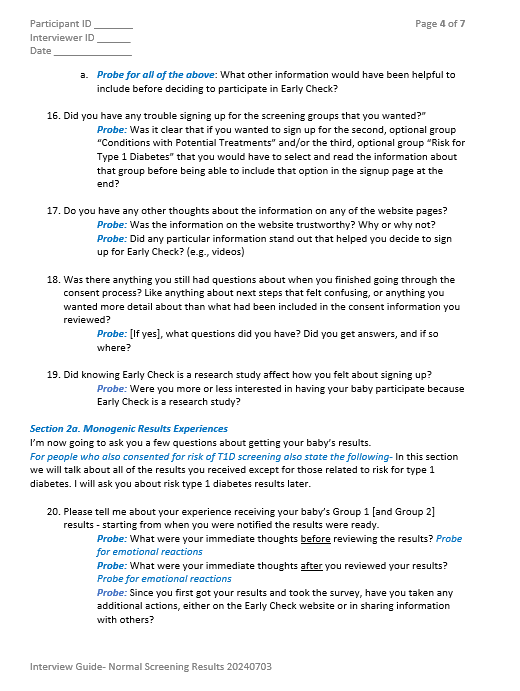

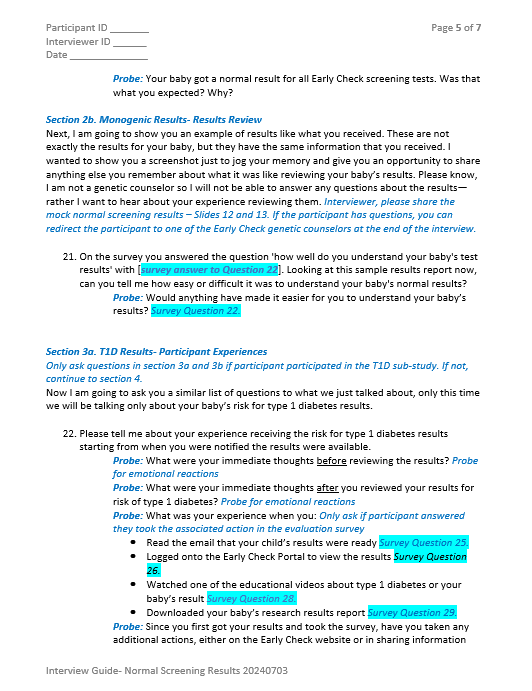

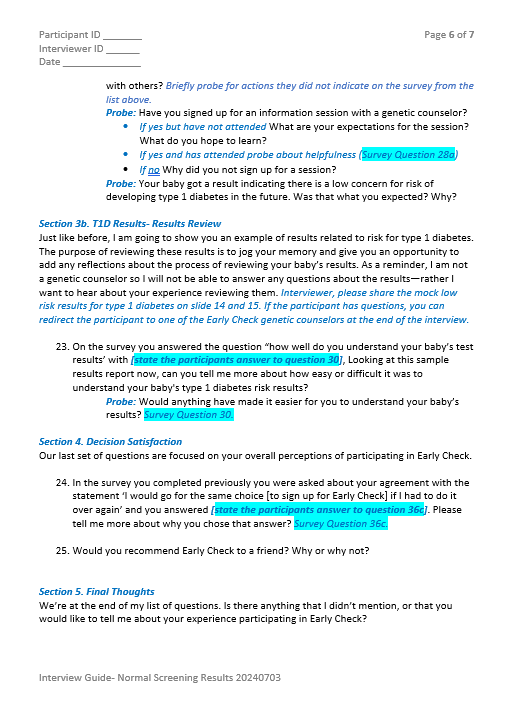

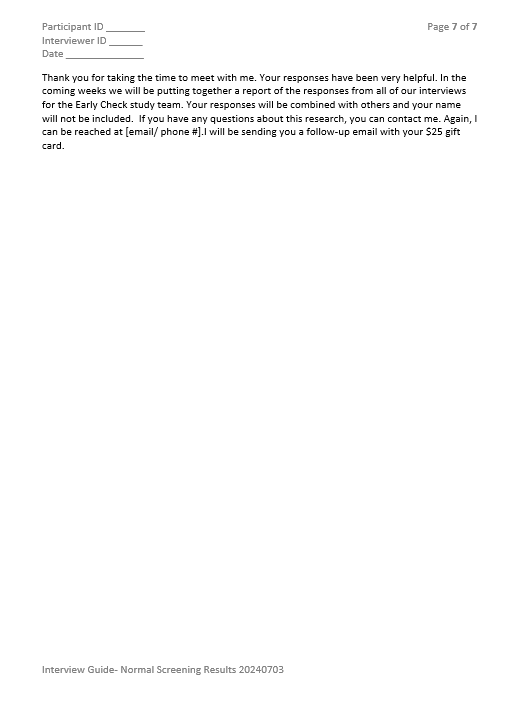

Supplement: Supplementary file 1 [file DataSheet2.docx]

**
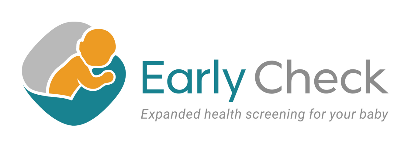
Supplementary File 1. Survey for Screened Negative Participants**


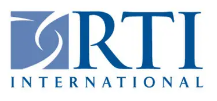


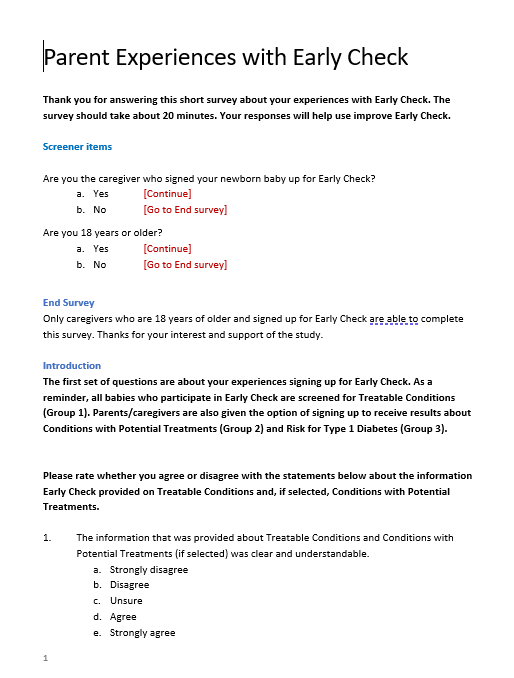


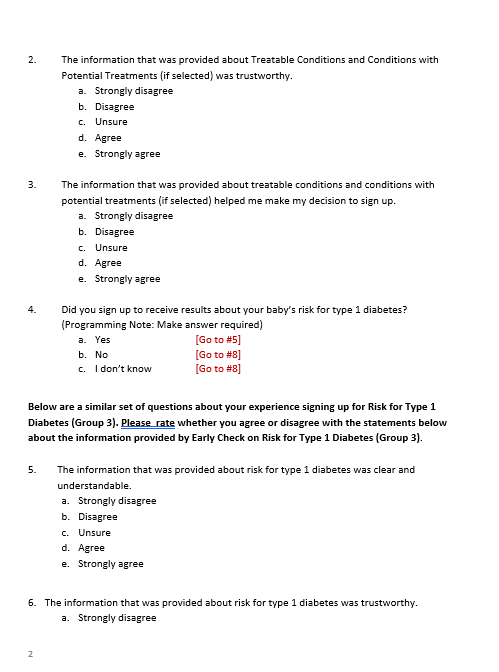

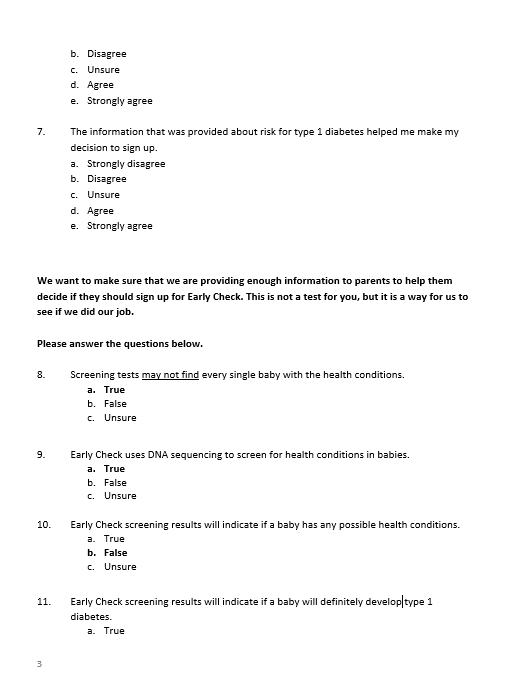

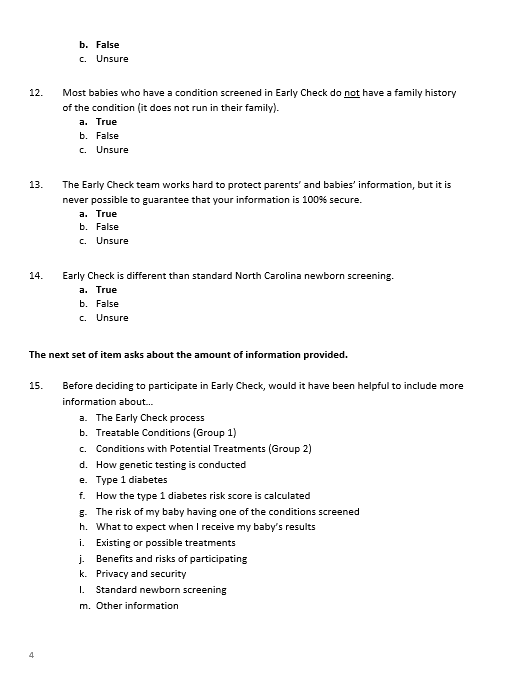

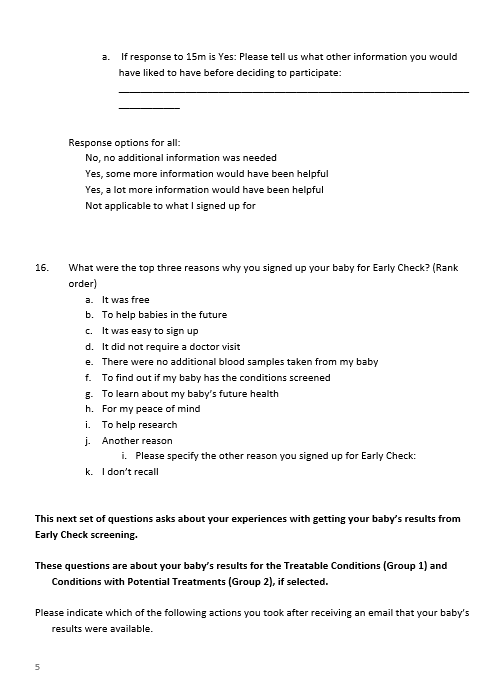

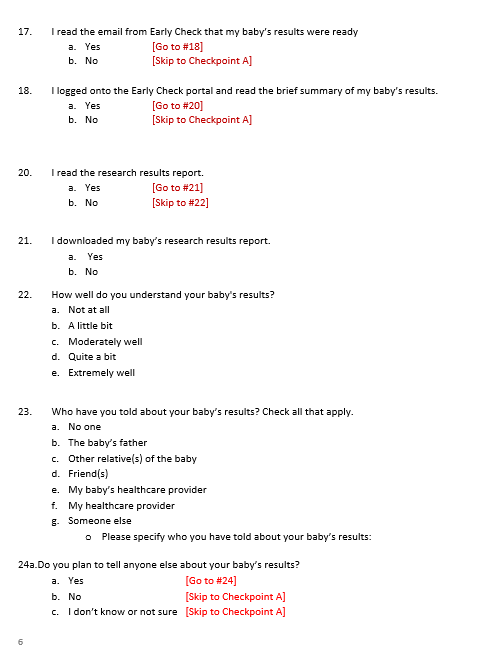

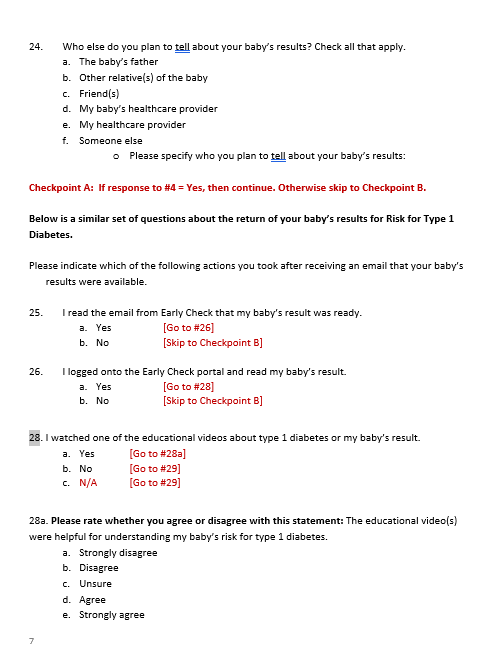

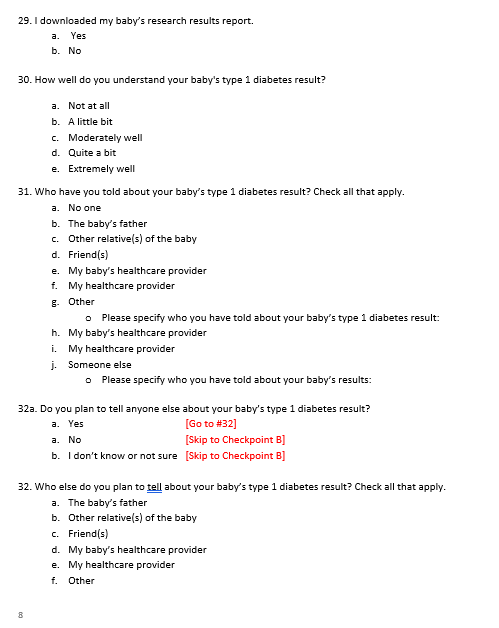

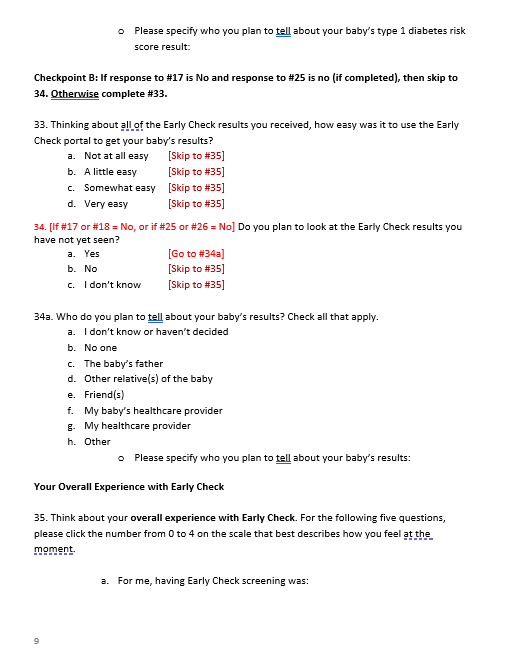

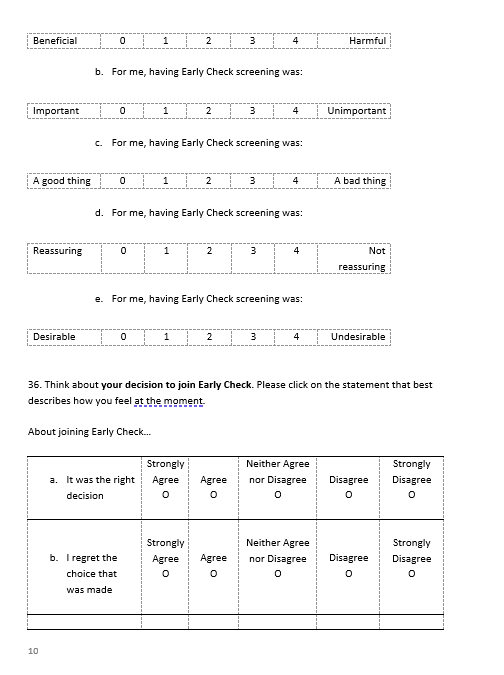

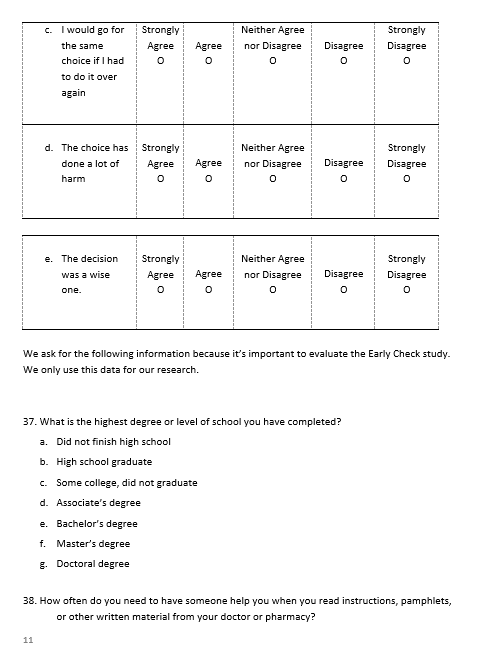

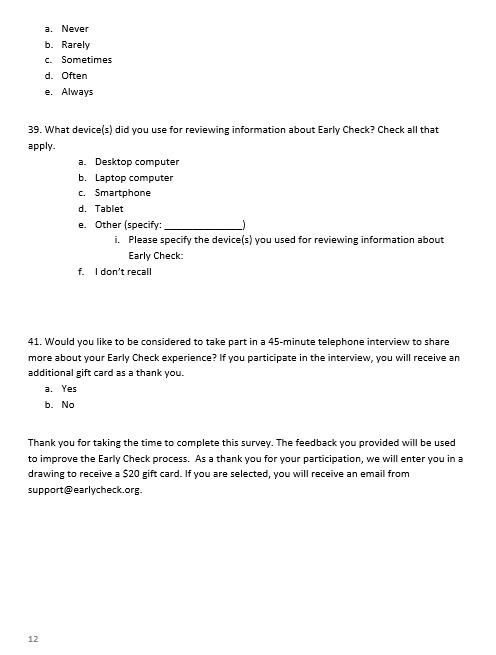

Supplement: Supplementary file 2 [file DataSheet1.docx]
